# A Terahertz Detector Based on Double-Channel GaN/AlGaN High Electronic Mobility Transistor

**DOI:** 10.3390/ma14206193

**Published:** 2021-10-18

**Authors:** Qingzhi Meng, Qijing Lin, Feng Han, Weixuan Jing, Yangtao Wang, Zhuangde Jiang

**Affiliations:** 1 State Key Laboratory of Mechanical Manufacturing Systems Engineering, Xi’an Jiaotong University, Xi’an 710049, China; aaaa.aa@stu.xjtu.edu.cn (Q.M.); qjlin2015@xjtu.edu.cn (Q.L.); wxjing@mail.xjtu.edu.cn (W.J.); wzzf001@stu.xjtu.edu.cn (Y.W.); zdjiang@mail.xjtu.edu.cn (Z.J.); 2 Collaborative Innovation Center of High-End State Key Manufacturing Equipment, Xi’an Jiaotong University, Xi’an 710054, China; 3 School of Mechanical and Manufacturing Engineering, Xiamen Institute of Technology, Xiamen 361021, China; 4 Chongqing Technology and Business University, Nan’an District, Chongqing 400067, China

**Keywords:** terahertz detector, responsivity, noise equivalent power, double-channel, high-electron-mobility transistor

## Abstract

A double-channel (DC) GaN/AlGaN high-electron-mobility transistor (HEMT) as a terahertz (THz) detector at 315 GHz frequency is proposed and fabricated in this paper. The structure of the epitaxial layer material in the detector is optimized, and the performance of the GaN HEMT THz detector is improved. The maximum responsivity of 10 kV/W and minimum noise equivalent power (NEP) of 15.5 pW/Hz^0.5^ are obtained at the radiation frequency of 315 GHz. The results are comparable to and even more promising than the reported single-channel (SC) GaN HEMT detectors. The enhancement of THz response and the reduction of NEP of the DC GaN HEMT detector mainly results from the interaction of 2DEG in the upper and lower channels, which improves the self-mixing effect of the detector. The promising experimental results mean that the proposed DC GaN/AlGaN HEMT THz detector is capable of the practical applications of THz detection.

## 1. Introduction

Terahertz (THz) detection technology has played an important role in the fields of spectrum inspection, non-destructive imaging, space exploration, etc., in the past decades. Various types of THz detectors have been developed and brought into practical applications. Thermal THz detectors such as bolometers, pyroelectric detectors, and Golay cells have high sensitivity and ultra-wide detection bandwidth. However, their common disadvantages are the long response time and poor resistance to the thermal source in the environment. Since Dyakonov and Shur [1,2] proposed the plasma oscillation theory in field-effect transistors (FETs), THz detectors of FET types like metal oxide semiconductor (MOS) FETs [3,4,5,6], III–V high electron mobility transistor (HEMT) [7,8,9,10,11,12], and FETs based on two-dimensional materials [13,14,15,16,17,18] were intensively studied because of their fast response rate and micro-nano size. Among different types of FETs, GaN/AlGaN HEMTs received extensive attention for their excellent frequency and power characteristics, which are very suitable for operating in the THz region. During the past decades, GaN/AlGaN HEMT THz detectors have achieved impressive development. The minimum noise equivalent power (NEP) at room temperature was able to reach the order of 10 pW/Hz^0.5^ beyond 1 THz [7,8,9,10,11,12]. In addition, THz detectors based on HEMTs have been prepared in an array on chips and applied in real-time imaging [19,20,21]. However, most of the related work mainly focused on the optimization of THz antenna [7,8,10] and the structure of GaN HEMT [11,12]; rarely has novel epitaxial material structures of GaN HEMT THz detectors been reported. It was noticed that the epitaxial layer material property is not only related to the crystal quality but also affects the self-mixing effect of GaN/AlGaN HEMT THz detectors. Therefore, the improvement of the epitaxial layer material property will enhance the THz detector performance. In our previous work [22], we proposed a GaN/AlGaN DC HEMT THz detector and optimized its structure parameters by technology computer-aided design (TCAD) simulation software, which theoretically predicted that GaN/AlGaN DC HEMT could be used as a THz detector. Here, a DC GaN/AlGaN HEMT THz detector was prepared, and the responsivity R_v_ and noise equivalent power (NEP) were measured at 315 GHz THz radiation. The results were compared with the current reported SC GaN/AlGaN HEMT THz detectors.

## 2. Detection Mechanism for DC GaN/AlGaN HEMT Detector

**Figure 1:**
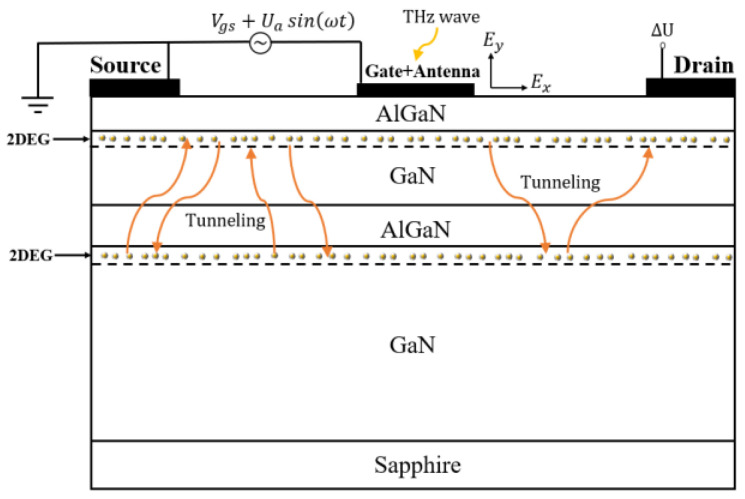
The schematic diagram of the operation mechanism of the DC GaN HEMT THz detector.

The cross-sectional structure of a typical DC GaN/AlGaN HEMT is shown in Figure 1. The 2DEG is formed at the upper and lower GaN/AlGaN heterojunction due to the polarization effect. THz radiation is coupled into the gate through an antenna and induces a small AC signal $U_{a}sin(\omega t)$, where *U*_a_ is the amplitude of the incident THz wave and *ω* is the angular frequency of the incident THz radiation. This perturbation will induce horizontal and perpendicular THz electric fields *E_x_* and *E_y_* to the channel. The 2DEG plasmon is then excited and a photocurrent/photovoltage signal is generated by the self-mixing effect. For the conventional SC GaN/AlGaN HEMT detector, the photocurrent is expressed as [11,12]:

(1) $$i=P_{0}Z_{0}\bar{z}\frac{dG_{0}\left(V_{gs}\right)}{dV_{gs}}\int_{0}^{L}\dot{E_{x}}\dot{E_{y}}\cos\varphi dx$$

where *P*_0_ is the power of incident THz, *Z*_0_ is the impedance of transmission free-space, $\bar{z}$ is the effective distance from the gate to the conductive channel, *G*_0_(*V_gs_*) is the channel conductance, and φ is the phase difference between *E_x_* and *E_y_*.

In low-temperature resonant detection mode, the resonant frequency follows the linear dispersion law:

(2) $$\omega =\frac{\pi s}{2L_{g}}\left(2n+1\right)\;n=0,\;1,\;2\cdots$$

where *ω* is the resonant frequency, *s* is wave velocity, and *L_g_* is gate length. The fundamental resonance frequency and its odd harmonics frequency depend on the gate length. Additionally, the distance between the two channels will influence the resonant intensity. When the separation of the two channels is smaller, the resonant intensity will increase because the coupling of the two channels is enhanced [23]. In room temperature non-resonant detection mode, the response signal of the detector is mainly influenced by the thickness of the barrier layer and the distance between two channels. The thicker barrier can supply larger 2DEG density while reducing the gate control capability [22]. The barrier height of the two channels is mainly related to the distance of the upper and lower channel. The smaller distance between the upper and lower channel, the lower the barrier height will be, leading to a greater tunnel probability. The design of this DC GaN HEMT THz detector is based on aforementioned parameters.

**Figure 2:**
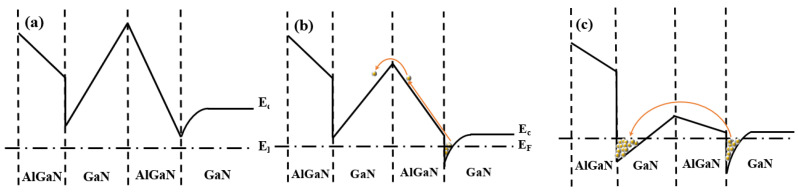
The band structure diagram of the GaN/AlGaN heterojunctions of (**a**) not contacted, (**b**) lower channel contacted, and (**c**) both channels contacted.

Figure 2 shows the energy band diagram at the top and the bottom heterojunction. When *V_gs_* is small, both the upper and lower channels are not conducted. With the increase of the *V_gs_*, the conduction band of the lower channel becomes lower than the Fermi level and the 2DEG is formed in the triangle barrier (Figure 2b). However, the barrier between the upper and lower channel is still too high. Only a small number of carriers can jump over the barrier and tunnel into the upper channel. When *V_gs_* continues to increase, both channels are conducted. The barrier between the upper and lower channel becomes lower. Thus, the tunneling probability increases and more carriers transform from the lower channel to the upper channel. Under this situation, a vertical built-in electric field from up to down is formed between the two channels, which leads to an increase of the perpendicular electric fields *E_y_* and the total THz response of the detector. 

## 3. Design and Experimental Procedure

**Figure 3:**
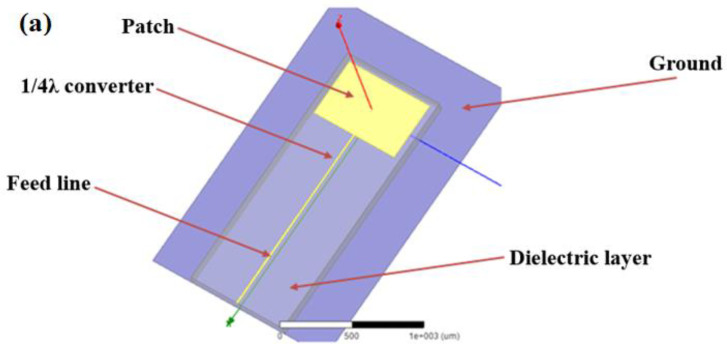

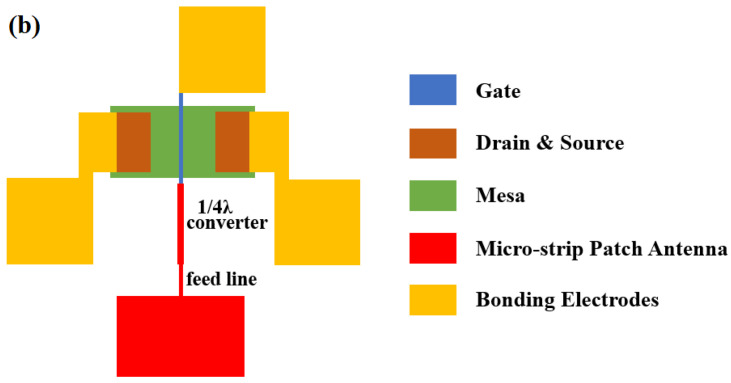
(**a**) The diagram of the micro-strip patch antenna, (**b**) the overall top view diagram of the GaN HEMT THz detector.

In our previous work [22], we designed the dimensions of the GaN HEMT detector including the epitaxial layer structure and gate length. In this paper, we designed a microstrip patch antenna with a center frequency of 315 GHz with a high-frequency structure simulator (HFSS). Figure 3a shows the diagram of the micro-strip patch antenna, and Figure 3b shows the overall top view diagram of the GaN HEMT THz detector. 

The patch antenna and the gate electrode of the GaN HEMT are connected by a feedline and a 1/4 λ impedance converter. The dimensions of the patch can be estimated as:

(3) $$W_{a}=\frac{c}{f\sqrt{2\varepsilon_{r}+2}}$$

(4) $$L=\frac{c}{2f\sqrt{\varepsilon_{e}}}-2\Delta L$$

(5) $$\Delta L=0.412h\left(\frac{\varepsilon_{e}+0.3}{\varepsilon_{e}-0.258}\right)\left(\frac{W_{a}/h+0.264}{W_{a}/h+0.8}\right)$$

where *W_a_* is patch width, *L* is patch length, *c* is light speed, *f* is oscillation frequency, *ε_r_* is the dielectric constant of the sapphire substrate, *ε_e_* is the effective dielectric constant of the sapphire substrate, and *h* is the thickness of the sapphire substrate (GaN/AlGaN thickness is ignored). As the feedline is directly connected with the gate, the impedance of the feedline should match the impedance of gate to source: $Z_{f}=\left|Z_{gs}\right|$, where *Z_f_* represents the impedance of the feedline, and $\left|Z_{gs}\right|$ is the magnitude of gate to source impedance. Based on the optimized device structure parameters in Ref. [22], the impedance of gate to source simulated by Silvaco is $Z_{gs}$ = 71.35 − j69.52 Ω, and the corresponding *Z_f_* equals 99.6 Ω. Then the width of the feedline *W_f_* is calculated as:

(6) $$Z_{f}=\{\begin{array}{c} \frac{60}{\sqrt{\varepsilon_{e}}}In\left(\frac{8h}{W_{f}}+\frac{W_{f}}{4h}\right)\;\;\;\;\;\;\frac{W_{f}}{h}\leq 1\\ \frac{120\varepsilon_{r}}{\sqrt{\varepsilon_{e}}\left[W_{f}/h+1.393+0.667In\left(W_{f}/h+1.444\right)\right]}\;\;\frac{W_{f}}{h}>1 \end{array}$$

**Figure 4:**
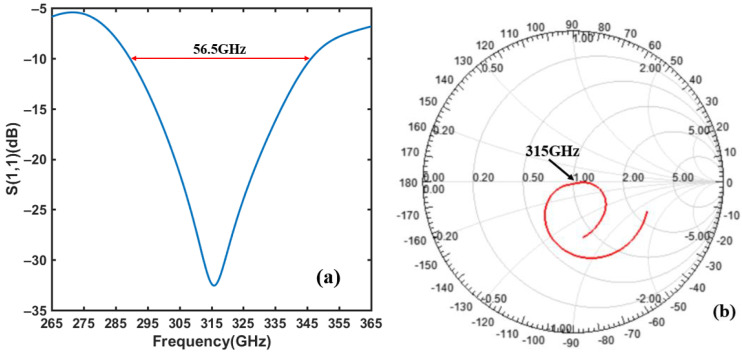
(**a**) Simulation result of the S(1,1) parameter for the microstrip patch antenna. (**b**) Smith chart for the impedance matching characteristics between the microstrip patch antenna and the gate electrode.

The typical 1/4 *λ* impedance converter is used for the impedance matching of the feedline and the patch antenna. The relationship between the impedance and dimensions of the patch antenna is as follows:

(7) $$Y_{in}\left(Z\right)=\frac{2G}{\cos^{2}\left(\beta z\right)}$$

(8) $$\beta =\frac{2\pi \sqrt{\varepsilon_{e}}}{\lambda_{0}}$$

(9) $$G=\{\begin{array}{c} \frac{1}{90}{\left(\frac{a}{\lambda_{0}}\right)}^{2}\;\;(a<0.35\lambda_{0})\\ \frac{1}{120}\frac{a}{\lambda_{0}}-\frac{1}{60\pi^{2}}\;(0.35\lambda_{0}\leq a<2\lambda_{0})\\ \frac{1}{120}\frac{a}{\lambda_{0}}\;\;\;\left(a\geq 2\lambda_{0}\right) \end{array}$$

where *z* is the distance between the feed point and the edge of the antenna. In this design, the central feeding mode is used (*z* = *W_a_*/2), and the impedance of the 1/*λ* converter is $Z_{0}=\sqrt{Z_{a}Z_{f}}$. The width of 1/4 *λ* converter *W*_0_ is obtained from Equation (4). Combining the calculation results and HFSS simulation optimization, the design parameters of the THz antenna are summarized in Table 1. The simulation results of S (1,1) parameters for the center frequency of 315 GHz antenna are shown in Figure 4a. The minimum S (1,1) parameter is as low as −32.7 dB, and the bandwidth is 56.5 GHz. As observed from the Smith chart in Figure 4b, the 315 GHz frequency point is located almost at the center of the chart, which indicates that the impedance of the antenna and the detector are well matched.

**Figure 5:**
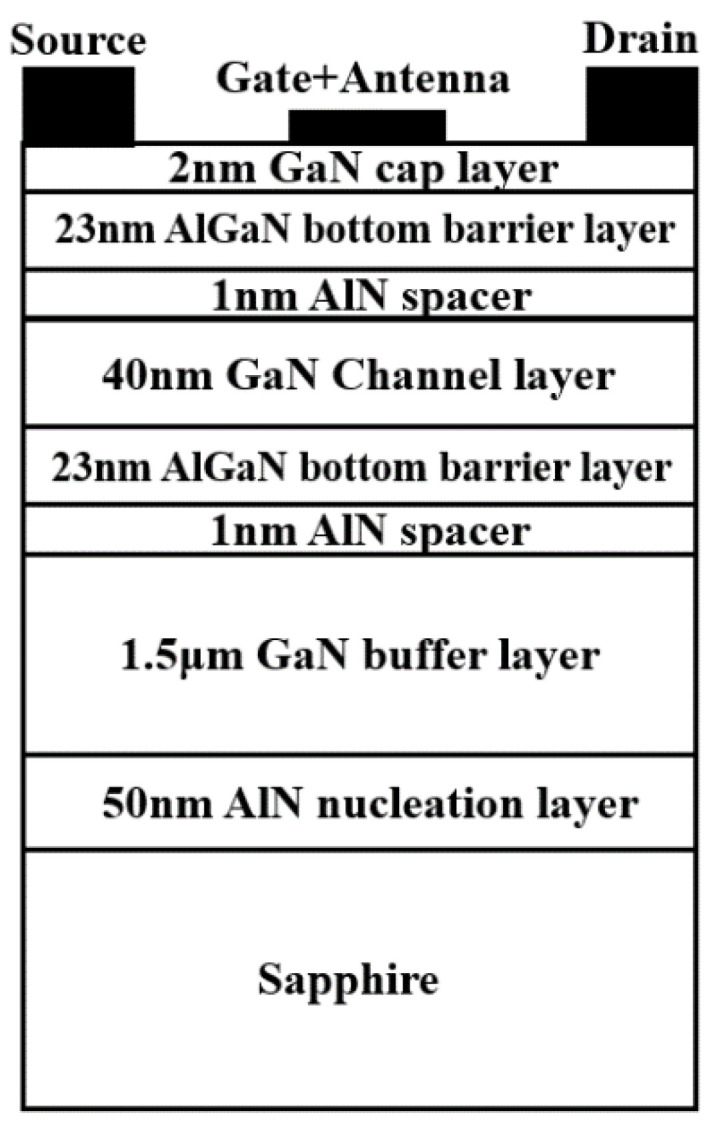
The cross-sectional diagram of the prepared DC GaN HEMT THz detector.

**Figure 6:**
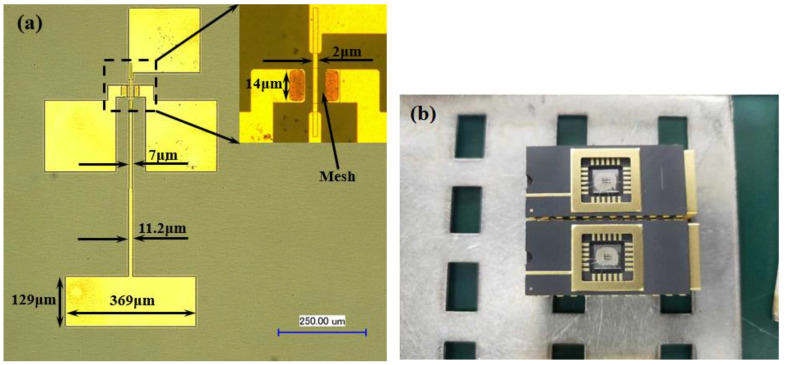
(**a**) Top view of the prepared DC GaN HEMT detector integrated with microstrip patch antenna. (**b**) THz chips packaged by DIP24 ceramic shell.

The deposition of the DC GaN/AlGaN epitaxial layer material was carried out by the metal-organic chemical vapor deposition (MOCVD) method. A 50 nm AlN nucleation layer was deposited on the sapphire substrate followed by a 1.5 μm GaN buffer layer, 1 nm AlN spacer, 23 nm Al_0.25_Ga_0.75_N interlayer, 40 nm GaN channel layer, 1 nm AlN spacer, 23 nm Al_0.25_Ga_0.75_N barrier layer, and 2 nm GaN cap layer. The average electron mobility measured by the non-contact Hall measurement system is 1815 cm^2^/V·s^−1^, and the C-V measurement shows that the sheet density is 8.247 × 10^12^/cm^2^. The isolation mesa was etched by inductively coupled plasma (ICP) etching using BCl_3_/Cl_2_. The drain and source electrode were deposited with Ti/Al/Ni/Au (20 nm/120 nm/40 nm/50 nm) followed by 850 °C rapid annealing in N_2_ environment. The specific contact resistance evaluated by transmission line model measurement was 10^−6^ Ω·cm^2^. The gate electrode was made of Ni/Au (30 nm/80 nm) through UV-lithography and the lift-off process. The microstrip antenna and bonding electrode were deposited with Ni/Au 20 nm/200 nm. The substrate was then thinned down to 155 μm, and a 1 μm thickness Cu film was deposited on the back of the substrate as the group plate of the antenna. The cross-sectional diagram of the prepared DC GaN HEMT THz detector is shown in Figure 5. Finally, the prepared THz chip was packaged into a DIP24 ceramic shell. The optical microscope image for the top view of the prepared detector is shown in Figure 6a, and the packaged chips of the detectors are shown in Figure 6b.

**Figure 7:**
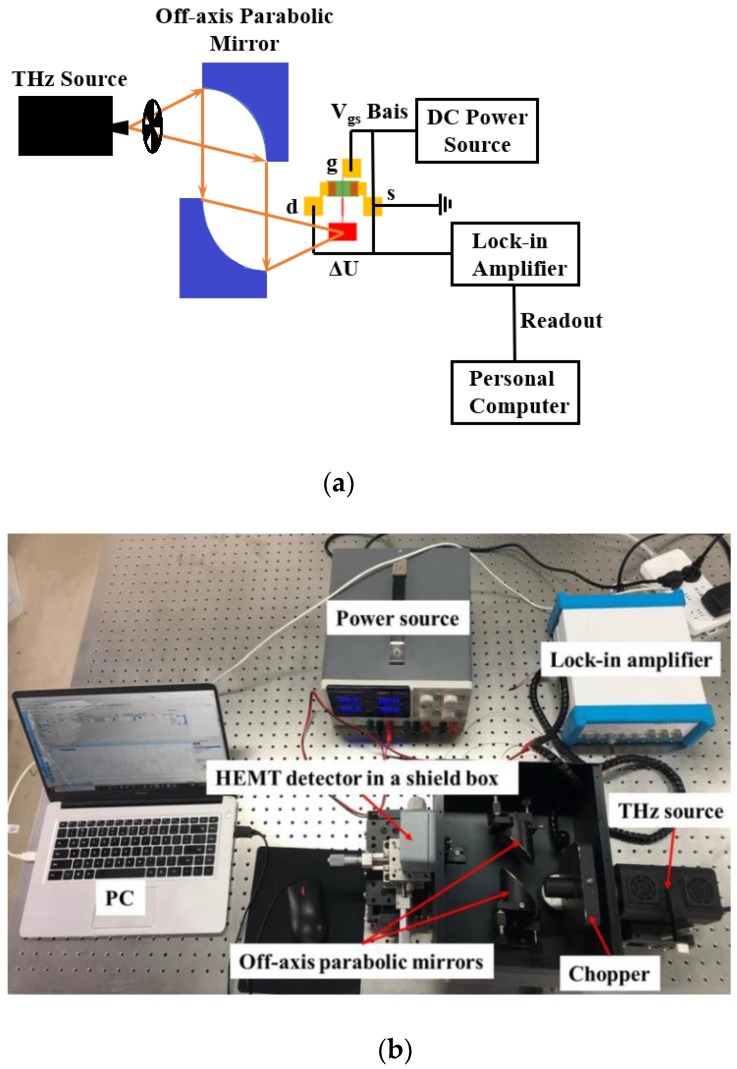
The platform for the characterization of the detector: (**a**) the diagram; (**b**) the practical picture.

Figure 7 shows the schematic of the platform for the characterization of the detector. Continuous THz radiation was generated from an IMPATT diode 315 GHz frequency THz source and modulated by an SDC-500 chopper. The modulated THz wave was then collimated and focused onto the DC HEMT detector by two 2-inch diameter off-axis parabolic mirrors. An MFLI-5M lock-in amplifier was used for the measurement of the photoinduced drain to source voltage Δ*U* (the source side of the device is grounded), and the measured Δ*U* was read out by a personal computer (PC). The *V_gs_* bias of the detector is supplied by a UTP 3305 DC power source. A Golay cell detector was used for the calibration of the incident THz power.

## 4. Results and Discussion

**Figure 8:**
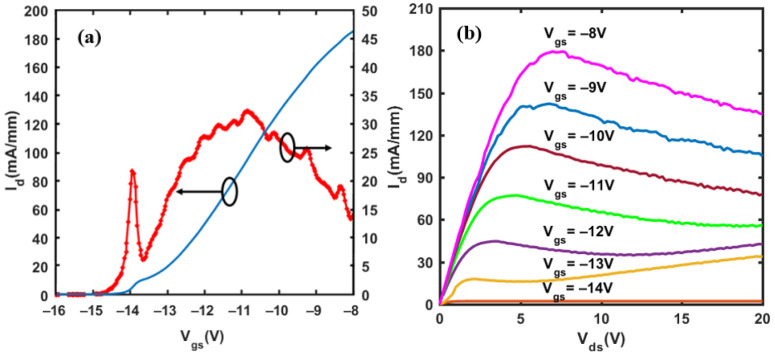
(**a**) The transfer and transconductance characteristics of the fabricated DC GaN HEMT. (**b**) The output characteristics of the fabricated DC GaN HEMT.

The direct-current characteristics of the DC GaN/AlGaN HEMT are tested by B1500A Semiconductor Device Analyzer. The results of transfer and output characteristics are shown in Figure 8a,b, respectively. There are two peaks in the transconductance characteristic curve (Figure 8a) corresponding to the upper and lower channel of the HEMT. The two peak values of gm are 22 mS/mm at *V_gs_* = −14 V and 33 mS/mm at *V_gs_* = −11 V, respectively, showing a good gate control ability. The output characteristics in Figure 8b show that the saturation output current reaches 180 mA/mm. Due to self-heating and the low thermal conductivity of the sapphire substrate, the HEMT has a current collapse effect, which makes the drain current slightly decrease in the saturation region. The results of the DC performance of the DC GaN/AlGaN HEMT show that the prepared device qualifies as a THz detector.

**Figure 9:**
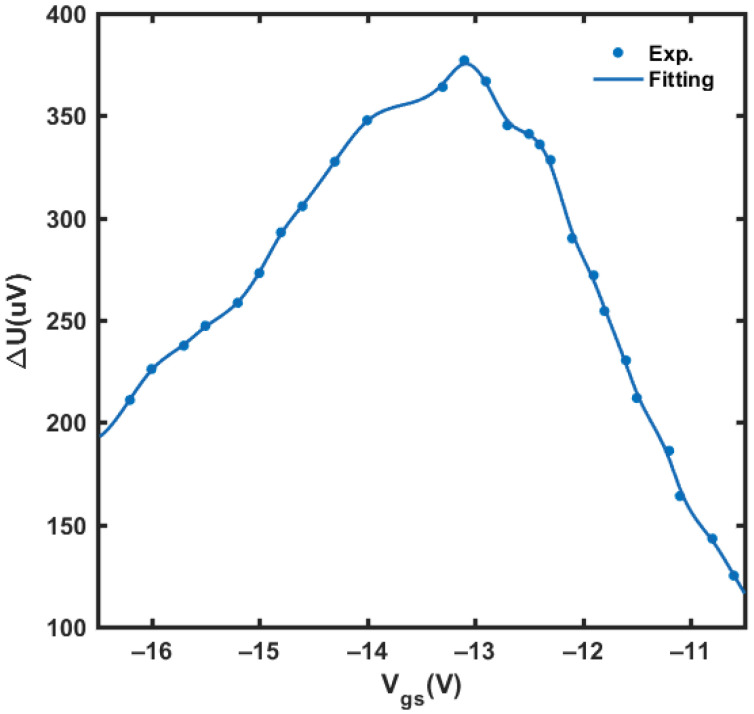
The response voltage Δ*U* with the variation of gate-source bias *V_gs_*.

The two main parameters of the detector, voltage responsivity *R_v_* and NEP, were characterized by the experimental setup described in Section 3. The experimental results of Δ*U* with the change of gate-source bias *V_gs_* are shown in Figure 9. The maximum response voltage of 377 μV at 1025 Hz modulation frequency is obtained at *V_gs_* = −13.1 V without any external amplifier circuit.

**Figure 10:**
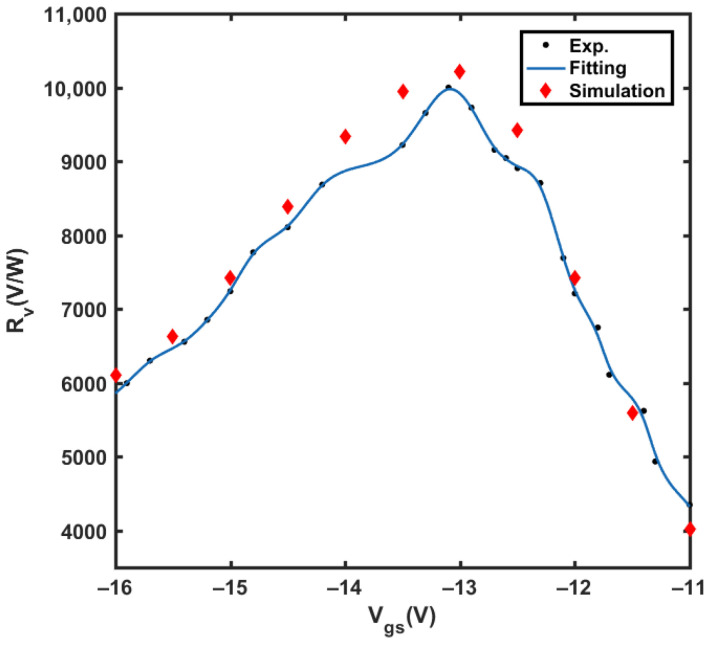
The experimental and simulation results of responsivity with the change of *V_gs_*.

**Figure 11:**
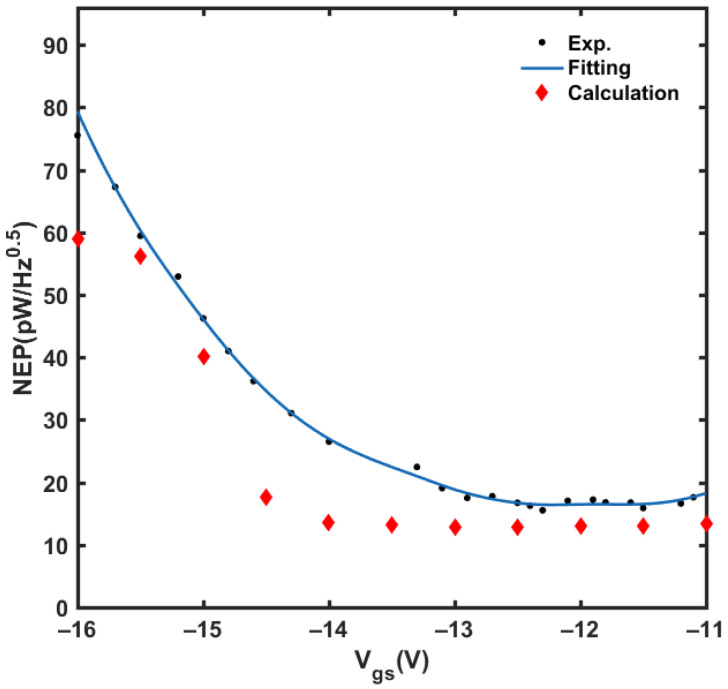
The experimental and simulation results of NEP with the change of *V_gs_*.

The *R_v_* can be calculated from the measured Δ*U* by Equation (10):

(10) $$R_{v}=\frac{\Delta US_{t}}{PS_{e}}\frac{\pi }{\sqrt{2}}$$

where *S_t_* is the total area of the THz beam spot, *S_e_* is the effective area of the detector, *P* is the total THz radiation power on the beam spot, and factor $\frac{\pi }{\sqrt{2}}$ originates from the rms amplitude in the lock-in amplifier and the Fourier transform of square wave modulated signal. The effective area of the detector can be estimated using the “antenna gain” method [24]:

(11) $$S_{e}=\frac{G\lambda^{2}}{4\pi }$$

where *G* is antenna gain, and λ is the wavelength of the incoming THz wave. The value of *G* = 1.80 is simulated by HFSS and *λ* = 952 μm. In our experiment, *S_t_* is measured as π × (3 mm)^2^ by a THz detection card, and the THz radiation power calibrated by the Golay cell detector is 19 μW. The comparison between the experimental and simulation results is shown in Figure 10. The method and models used in the simulation are described in [22]. It is seen that the peak responsivity reaches 10 kV/W, and the experimental results are consistent with the simulation results. The NEP of the detector is expressed as Equation (12):

NEP = *V_n_*/*R_v_* (12)

where *V_n_* is the background noise voltage. The measurement and theoretical calculation results of NEP are shown in Figure 11, and the minimum NEP measured is 15.5 pW/Hz^0.5^. It can be seen that the measurement results of NEP are higher than the calculated results. In practical measurement, the background noise is directly measured by the lock-in amplifier, which includes the thermal noise in the GaN HEMT, the 1/f noise, and even the electromagnetic interference in the environment. In the theoretical calculation, *V_n_* only contains the thermal noise $\sqrt{4kTR_{ds}}$, where *R_ds_* is the channel resistance extracted from the output characteristic curve.

Table 2 shows the performance comparison of different types of SC GaN/AlGaN HEMT THz detectors in recent years. Our DC GaN/AlGaN HEMT THz detector is superior to most of the reported SC detectors in terms of NEP. In addition, it is comparable to the existing detectors with the best performance. The excellent performance of the prepared detector mainly results from the enhancement of the mixing effect in the DC channel. We did not use an external amplifier circuit or gain elements such as a waveguide and Fabry-Perot cavity. It is believed that the NEP of our DC GaN HEMT THz detector could be reduced down to 1 pW/Hz^0.5^ if the detection system and the signal process method are further optimized. All these results demonstrate that the proposed DC GaN/AlGaN HEMT THz detector has potential value in the practical application of THz detection.

## 5. Conclusions

In this paper, a DC GaN HEMT THz detector at the detection frequency of 315 GHz is proposed. The THz microstrip patch antenna is designed by HFSS simulation software, and the electrical performance and THz detection performance of the detector are characterized. The maximum voltage responsivity reaches 10 kV/W, and the minimum NEP is as low as 15.5 pW/Hz^0.5^. Experimental results demonstrate that the performance of the proposed DC GaN HEMT THz detector is more promising than most of the current SC GaN HEMT THz detectors, which means they are more capable for practical applications.

## Figures and Tables

**Table 1:** The design parameters of a THz antenna.

Central Frequency (GHz)	*W_a_* (μm)	*L_a_* (μm)	*W*_0_ (μm)	*W_f_* (μm)
315	369	129	7	11.2

**Table 2:** Comparison of different types of GaN/AlGaN HEMT THz detectors.

Structure Types	Detection Frequency (THz)	NEP (pW/Hz^0.5^)	Reference
Bow-tie & Si lens	0.5–0.64	25–40	[9]
Bow-tie & Fluorine ion implantation	0.65	47	[11]
Waveguide port horn antenna	0.29–0.36	76	[10]
Fabry-Perot cavity	0.14	4.26	[7]
Nano antenna	0.14	0.58	[8]
This work	0.315	15.5	

## Data Availability

The data presented in this study are available on request from the corresponding author.
